# Do work and family care histories predict health in older women?

**DOI:** 10.1093/eurpub/ckx128

**Published:** 2017-09-23

**Authors:** Rebecca Benson, Karen Glaser, Laurie M. Corna, Loretta G. Platts, Giorgio Di Gessa, Diana Worts, Debora Price, Peggy McDonough, Amanda Sacker

**Affiliations:** 1 Department of Epidemiology and Public Health, University College London, London, UK; 2 Department of Global Health and Social Medicine, Institute of Gerontology, Faculty of Social Science and Public Policy, King’s College London, The Strand, London, UK; 3 Stress Research Institute, Stockholm University, Stockholm, Sweden; 4 Department of Social Policy, The London School of Economics and Political Science, London, UK; 5 Dalla Lana School of Public Health, University of Toronto, Toronto, ON, Canada; 6 Manchester Institute for Collaborative Research on Ageing, University of Manchester, Manchester, UK

## Abstract

**Background:**

Social and policy changes in the last several decades have increased women’s options for combining paid work with family care. We explored whether specific combinations of work and family care over the lifecourse are associated with variations in women’s later life health.

**Methods:**

We used sequence analysis to group women in the English Longitudinal Study of Ageing according to their work histories and fertility. Using logistic regression, we tested for group differences in later life disability, depressive symptomology and mortality, while controlling for childhood health and socioeconomic position and a range of adult socio-economic circumstances and health behaviours.

**Results:**

Women who transitioned from family care to either part-time work after a short break from the labour force, or to full-time work, reported lower odds of having a disability compared with the reference group of women with children who were mostly employed full-time throughout. Women who shifted from family care to part-time work after a long career break had lower odds of mortality than the reference group. Depressive symptoms were not associated with women’s work and family care histories.

**Conclusion:**

Women’s work histories are predictive of their later life disability and mortality. This relationship may be useful in targeting interventions aimed at improving later life health. Further research is necessary to explore the mechanisms linking certain work histories to poorer later life health and to design interventions for those affected.

## Introduction

As increasing numbers of people live longer in old age in high income countries, demands on health and social services are likely to increase since the prevalence of chronic diseases and disabilities rises with age. Relieving these pressures will require improving the health of older adults more generally. This will in turn require an understanding of risk factors from earlier in the life course in order to identify those at subsequent risk of ill-health or disability in later life.

Paid work has been documented convincingly as an important determinant of health.^1–3^ Over the past few decades, social and policy changes have increased women’s participation in paid work and allowed for a variety of ways of combining work with family life. However, women’s working lives often remain constrained by family responsibilities.^4^^,^^5^ These forces have the potential to shape women’s longer term health. The demands of work and parenting may be in conflict and could result in health and self-care being given a lower priority—the multiple burden or role conflict hypothesis.^6^ Alternatively, combining parenting and paid work may contribute a health advantage—the role enhancement hypothesis.^7–9^

The way an individual combines work with family care is dynamic, and women employ different strategies to combine work and family roles over the life course. Thus, static, single time-point measures are unlikely to capture circumstances well. Although this has been recognized in recent research which takes advantage of the opportunities offered by sequence analysis to study the relationships between life course patterns and health,^10–17^ only a few studies have considered health in later life when paid work and intensive family care responsibilities may have ended. Depending on the health outcome (e.g. self-rated health, mortality or frailty), the methods used to identify and summarize lifecourse patterns, and the country considered (USA or England), these studies have reached varying conclusions on whether work–family histories predict differences in later life health.^10^^,^^13^^,^^17^ Lu et al*.*,^17^ examining frailty trajectories among English women aged 60+, reported beneficial effects for those who took a short career break before returning to work part-time compared with women who worked mostly full-time throughout (FTT). In American mothers aged 55+, Sabbath et al.^13^ found lower mortality in those who returned to work after briefly staying home with children compared with non-workers, always workers and those who delayed work re-entry. In contrast, Stone et al.^10^ found that English women aged 64+ did not differ in their odds of poor self-rated health according to their work–family histories.

In addition to the somewhat conflicting results of extant studies, previous research linking work histories to later life health has not considered disability or depression. Disability is an important indicator of health at older ages because it is associated with reduced quality of life^18^ and is responsible for a substantial component of the health and social care costs of ageing populations.^19^ Depression is similarly associated with diminished quality of life^20^ and increased use of health care.^21^ We fill this gap, as well as adding to evidence from previous research on mortality in American women, using data from a sample of English women with work histories recorded from ages 16 to 59. Using ideal-type sequence analysis of work histories and identifying mothers from fertility histories, we summarized nine patterns of combining work with family care across the lifecourse. We investigate whether there are differences in disability, depression or mortality according to these histories, while taking into account early life health, social position and other socio-economic factors.

## Methods

Data come from the English Longitudinal Study of Ageing (ELSA).^22^ ELSA began in 2002 as a longitudinal study of the population aged 50 years and older.^23^ Refreshment samples have been added to maintain the study’s representativeness of the over-50 population. Interviews are conducted with respondents biennially, and in wave three (2006/07) respondents provided retrospective histories from age 16 of their employment and family life, i.e. partnership and parenthood status. We first limited the sample to women who had reached state pension age (60 years or older^24^) at the time work and family histories were recorded. To limit heterogeneity due to changes in work and family life norms for women over the course of the twentieth century, we applied an upper age limit of 75 to our analyses of disability and depressive symptoms, and a lower age limit of 76 to our study of mortality. Information on the dependent variables was taken from waves 1 to 3 (in the case of disability and depressive symptoms) and wave 3 to February 2012 (in the case of mortality). Our complete-case sample was 2441 women, out of 2718 age-eligible women whose data were collected at the life history interview.

### Dependent variables

We used three variables to measure health in later life: disability, depression and mortality. Participant reports of difficulties with any activities of daily living (ADL: dressing, walking across a room, bathing, eating, getting in and out of bed, and using a toilet),^25^ or difficulties with any instrumental activities of daily living (IADL: preparing a hot meal, grocery shopping, using a telephone, managing medications and managing money)^26^ were used to create the disability measure. Specifically, a binary measure of disability was created for reports of difficulties with any of the ADL or IADL items. We used disability measured as soon after participants reached state pension age as possible. For those who were 60 years or older at the inception of ELSA—the majority of participants—this was wave 1, while for those who reached 60 years of age between wave 1 and the collection of work histories, this was waves 2 or 3.

Depression was measured using the Centre for Epidemiologic Studies Depression Scale, a validated measure for assessing depressive symptoms in older populations.^27^ Participants were asked whether or not they had experienced each of eight symptoms in the previous week, and, in line with previous studies, those who reported four or more symptoms were categorized as at risk of depression.^28^^,^^29^ Like disability, depression was measured as soon after respondents reached 60 years of age as possible.

Mortality data in ELSA come from record linkage to National Health Service data when participants have given their consent for such linkage. The mortality follow-up period ran from wave three (2006/07) when work histories were collected to February 2012, after which no updated mortality data were available at the time of this research.

### Independent variable

A year-by-year history of each woman’s labour market activities between the ages of 16 and 59 was created, primarily using information from the life history interview collected between waves 3 and 4 and supplemented with data from waves 1 to 6. Gaps in women’s work histories were filled using multiple imputation, with 20 imputed datasets created. It is possible that the work histories may be affected by inaccurate recall of events 40 or more years ago. However, many of the transitions in which we are interested, such as workforce entry or exit and changes from full- to part-time hours, will coincide with major milestones in the lives of sample members and their families, such as the birth of a child or a child starting school. Because the timing of these milestones is likely to be recalled accurately, the associated employment transitions may also be more accurately reported than long ago events not associated with an easily remembered milestone. To the degree that participants’ recollections are inaccurate, the most likely result is random measurement error which could bias our findings towards the null.

Optimal matching analysis grouped respondents in each dataset according to their patterns between ages 16 and 59 of working full-time, working part-time, or not performing any economic activity. Specifically, each woman’s work history was compared against a typology or set of ideal-types and assigned to one of these patterns by a process of dynamic hamming. Further details of the derivation of the ideal-types and the assignment of respondents to them are available elsewhere.^30^ Briefly, eight authors independently created ideal types, informed by theory and historical demographic data. There was substantial overlap between these sets of ideal types, and the final groupings were chosen by consensus. Where assignment differed between imputed datasets, the modal assignment for each respondent was used for analyses.

The seven-category work history typology contained the following categories: employed mostly FTT, mostly non-employed throughout (NET), early exit at about age 48, family carer to part-time employment following either a long career break from about ages 26–41 or a short career break from about ages 26 or 30, family carer to full-time employment following a medium career break from about ages 26 to 34, and mostly part-time throughout (PTT). It should be noted that individuals in each of the seven groups are mostly/always employed or non-employed around the specified ages because cases are matched to their closest model sequence and many actual sequences will not match exactly in every detail.

An important determinant of women’s trajectories is their parental status which is also associated with later life health.^31^ For this reason, we split the first two groups, FTT and NET, into those who ever had dependent children at home and those who did not, resulting in nine work–family histories for analysis. Small cell sizes precluded further differentiation of other trajectories.

### Covariates

Our analyses control for age, childhood health, childhood social class, smoking status, household wealth and education, which are all potential confounders of the relationship between the work history groups and later life health.^32–34^ We also control for age, using a series of dummies for ages 60, 61, 62 and so on up to age 90. Respondents reported their childhood health retrospectively in wave three using a five-point scale ranging from excellent to poor; this was dichotomized into excellent, very good or good versus fair or poor. Childhood social class, reported in wave three, was based on father’s occupation when the respondent was age 14 and was collapsed into three categories: manual, service and professional.^35^ Education is a five-category variable (less than O level, O level or NVQ2, A level or NVQ3, any tertiary education including NVQ4 and above, and foreign qualifications). Smoking is a three-category variable (never smoker, former smoker and current smoker). Adult social class was measured by the three-level National Statistics Socio-economic Classification (NS-SEC)^36^ of the most recent job. NS-SEC classifications are based on the location in a system of authority and the economic security and opportunities for advancement of a given position and can be applied to the whole adult population, including those not currently working. Wealth, again measured concurrently with disability and depression, was assessed in quintiles and included savings, investments and housing net of mortgage and other debt. ELSA provides wealth information at the level of ‘benefit unit’ which is defined as a couple or a single person along with any dependent children.^37^ Marital status, measured in the same wave as disability and depressive symptoms, was distinguished as currently married/partnered, never married and previously married (i.e. divorced or widowed).

For all three outcomes we fit three logistic models. The first model adjusted only for age. The second model also included potential confounders from childhood, and the third model added confounders from adulthood.

## Results

Characteristics of the sample are presented in Supplementary table S1 and Supplementary figures S1–S3. Of the sample, 28.9% reported disability, 30.7% were at risk of depression and 11.0% died during the follow-up period. Women who were NET and had no children, who had short career breaks, or who worked PTT were more likely to have fathers who were manual workers than the sample as a whole. Those who worked FTT and those who took moderate career breaks had the highest rates of tertiary qualifications. Women who worked FTT and had no children were more likely to have never been married and less likely to be either married or divorced than the sample overall. Those who worked either FTT or PTT had the highest rates of smoking. The distributions of wealth and childhood health were comparable across groups.

Table 1 examines the relative odds of disability. When compared with women with children working mostly FTT, women who took short and medium career breaks had lower odds of disability. In contrast, mothers in the mostly not-employed group had higher odds of disability than the reference group, mothers working FTT. The addition of covariates to the model had little effect on the estimates.

**Table 1:** Odds of having a disability (women aged 60–75)

	Model 1^a^		Model 2^b^		Model 3^c^	
	OR	95% CI	OR	95% CI	OR	95% CI
Mostly FTT, children	–Ref–					
Mostly FTT, no children	0.95	(0.57, 1.57)	0.92	(0.55, 1.54)	1.04	(0.58, 1.85)
Mostly NET, children	1.90	(1.30, 2.76)	1.85	(1.27, 2.70)	1.93	(1.29, 2.89)
Mostly NET, no children	1.21	(0.48, 3.04)	1.17	(0.46, 2.95)	1.44	(0.56, 3.70)
Weak attachment, early exit	1.58	(0.98, 2.55)	1.48	(0.91, 2.39)	1.56	(0.94, 2.58)
Family carer to part-time(long break: about 16 years)	0.68	(0.43, 1.09)	0.67	(0.42, 1.06)	0.78	(0.48, 1.28)
Family carer to part-time(short break: about 4 years)	0.52	(0.32, 0.84)	0.50	(0.31, 0.82)	0.57	(0.34, 0.94)
Family carer to full-time (about 10 year break)	0.47	(0.30, 0.73)	0.45	(0.29, 0.71)	0.48	(0.30, 0.77)
Full-time to part-time (at about age 23)	0.99	(0.56, 1.74)	0.91	(0.52, 1.62)	0.92	(0.51, 1.66)

Source: ELSA, waves 1–3, 2006–07.^22^

a Adjusted for age.

b Adjusted for age and childhood characteristics: socio-economic position and self-rated health at age 14.

c Adjusted for age, childhood characteristics, and adult characteristics: education, marital status, smoking status and wealth.

Table 2 shows the relative odds of having depressive symptoms. When compared with mothers working FTT, mothers who were mostly non-employed and women who took a long career break had higher odds of depressive symptoms. No group had reduced odds of depressive symptoms compared with the reference group. Again, most estimated ORs were comparable across models.

**Table 2:** Odds of depressive symptoms (women aged 60–75)

	Model 1^a^		Model 2^b^		Model 3^c^	
	OR	95% CI	OR	95% CI	OR	95% CI
Mostly FTT, children	–Ref–					
Mostly FTT, no children	1.15	(0.69, 1.94)	1.15	(0.68, 1.95)	1.39	(0.76, 2.51)
Mostly NET, children	1.89	(1.27, 2.79)	1.85	(1.24, 2.75)	1.91	(1.26, 2.91)
Mostly NET, no children	0.62	(0.20, 1.91)	0.53	(0.17, 1.69)	0.68	(0.21, 2.19)
Weak attachment, early exit	1.48	(0.89, 2.48)	1.35	(0.80, 2.28)	1.41	(0.82, 2.41)
Family carer to part-time(long break: about 16 years)	1.56	(1.01, 2.40)	1.54	(0.99, 2.39)	1.82	(1.15, 2.88)
Family carer to part-time(short break: about 4 years)	0.66	(0.41, 1.06)	0.64	(0.40, 1.03)	0.71	(0.43, 1.17)
Family carer to full-time (about 10 year break)	0.97	(0.64, 1.46)	0.95	(0.62, 1.45)	1.04	(0.67, 1.60)
Full-time to part-time (at about age 23)	2.15	(1.25, 3.68)	1.90	(1.09, 3.28)	1.89	(1.06, 3.35)

Source: ELSA, waves 1–3, 2006–07.^22^.

a Adjusted for age.

b Adjusted for age and childhood characteristics: socio-economic position and self-rated health at age 14.

c Adjusted for age, childhood characteristics, and adult characteristics: education, marital status, smoking status and wealth.

Table 3 shows the relative odds of mortality. In each model, women who took long career breaks had lower odds of death over the follow-up period than mothers who worked FTT. No other group had odds of mortality that differed significantly from the reference group.

**Table 3:** Odds of mortality (women aged 76+)

	Model 1^a^		Model 2^b^		Model 3^c^	
	OR	95% CI	OR	95% CI	OR	95% CI
Mostly FTT, children	–Ref–					
Mostly FTT, no children	1.00	(0.54, 1.88)	1.03	(0.55, 1.93)	0.59	(0.26, 1.33)
Mostly NET, children	0.87	(0.52, 1.46)	0.89	(0.53, 1.50)	0.99	(0.58, 1.70)
Mostly NET, no children	1.02	(0.39, 2.71)	1.06	(0.40, 2.84)	0.91	(0.31, 2.66)
Weak attachment, early exit	1.10	(0.45, 2.67)	1.09	(0.45, 2.67)	1.22	(0.48, 3.12)
Family carer to part-time(long break: about 16 years)	0.37	(0.19, 0.74)	0.38	(0.19, 0.75)	0.44	(0.22, 0.88)
Family carer to part-time(short break: about 4 years)	0.49	(0.22, 1.09)	0.48	(0.22, 1.07)	0.52	(0.23, 1.20)
Family carer to full-time (about 10 year break)	0.66	(0.36, 1.24)	0.68	(0.36, 1.27)	0.76	(0.40, 1.45)
Full-time to part-time (at about age 23)	1.66	(0.53, 5.27)	1.73	(0.55, 5.50)	2.20	(0.66, 7.34)

Source: ELSA, waves 1–3, 2006–07.^22^

a Adjusted for age.

b Adjusted for age and childhood characteristics: socio-economic position and self-rated health at age 14.

c Adjusted for age, childhood characteristics, and adult characteristics: education, marital status, smoking status and wealth.

**Figure 1:**
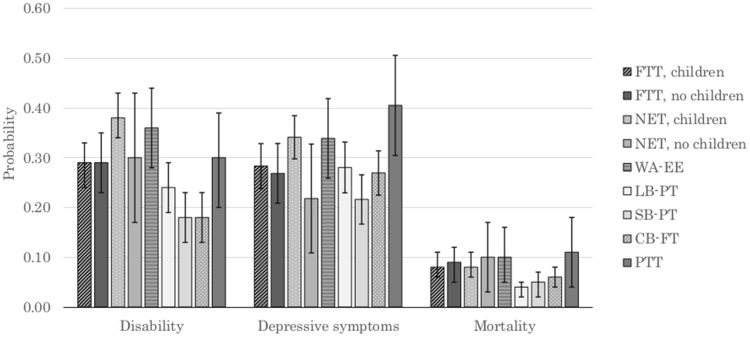
Probability of disability, depressive symptoms and death by work history. Sample for disability and depressive symptoms is aged 60–75 years; sample for mortality is aged 76+ years. FTT, mostly full-time throughout; NET, mostly non-employed throughout; WA-EE, weak attachment, early exit; LB-FT, family care to part- time, longer career break; SB-FT, family care to part time, shorter career break; CB-FT, family care to full-time, moderate career break; PTT, mostly part-time throughout. Error bars are 95% CIs

Figure 1 shows predicted probabilities of our three health endpoints by work history with age held at its mean, 70.7 years. The three career break groups had amongst the lowest probabilities for all three adverse outcomes. The highest probabilities of disability were found in mothers who were mostly non-employed and women who exited the labour market early, while those who transitioned from full- to part-time had the highest probability of depression. The greatest probability of mortality was observed for mostly non-employed women without children, women who exited the labour market early, and women who transitioned from part- to full-time work. The differences between the highest and lowest probabilities of disability and depression were statistically significant, but differences in mortality probabilities were not.

## Discussion

Women in this sample experienced a mixture of health advantages and disadvantages in later life if they reported spending some time caring for family and away from paid work as their main economic activity. Women who took short or medium career breaks had lower odds of disability in later life, while women who took longer career breaks had lower odds of mortality but higher odds of depressive symptoms. Conversely, those who reported family care as their main pursuit throughout the lifecourse to age 59 without much engagement in paid work encountered some health disadvantage.

Previous research has examined work histories and later life health in ELSA women. Our finding that women transitioning from family care to full-time work were less likely to report a disability contrasts with previous studies which found no difference in self-rated health between women who returned to work after career breaks and women who worked throughout.^10^ These distinctions may reflect differences in the outcomes or in the sequence analysis ideal-types. In a comparable cohort of American women, unmarried non-working mothers had the highest mortality rates, and these were significantly higher than those experienced by mothers who took career breaks.^13^ Although our results do not distinguish between unmarried and married women, we also found significantly lower odds of mortality in one group of mothers who took a career break.

Much previous work in this area has been framed by theory about the health effects of occupying multiple roles, with particular attention given to the role enhancement and role overload hypotheses. We find some support for role enhancement: mothers who were in full-time paid work throughout their lives had lower odds of later life disability than mothers who were exclusively family carers throughout their lives. Yet our other results do not support either hypothesis: there was no difference for any outcome between mothers who worked FTT and those without children who did the same. Role enhancement would predict the former to be in better health, while role overload would do so for the latter group. Further, it is not clear where groups defined by distinct periods of family care and periods of employment—who had lower odds of disability and mortality—fit within the role enhancement/role overload framework which does not consider roles as changing over the life course.

Further research is warranted into why mothers who were mostly non-employed and women who took long career breaks had higher odds of depressive symptoms. Because we do not have information on depression in childhood, it could be that depressive symptoms contributed to some women spending more time away from paid work. Alternatively, and in keeping with the role enhancement hypothesis, it may be that long periods focused solely on family care and/or long periods of not being in paid employment contribute to the development of depressive symptoms.

Our findings add to the accumulating evidence that women’s roles as paid workers and providers of family care through their early and mid-adult life are predictive of their later life health. In common with other observational studies, these findings do not necessarily reflect causal effects. One barrier to causal interpretation is that the least healthy members of the population are less likely to have reached the age requirements for entry to ELSA (age 50 years) or the age limits we impose for this study (60 years, to coincide with state pension age). This potential bias is exacerbated by the large age range in the sample; the oldest respondents are over 90-years old. Although we control for age, it is still possible that the healthy oldest survivors are distributed differently in the work–family history groups than were unobserved non-surviving members of their age cohort.

Another barrier to causal interpretation is individual heterogeneity. The reasons individuals choose particular combinations of work and family care may also be related to health. For example, women in poor health during early adulthood may be more likely to have poorer health in later life and be less likely to work outside the home. The possibility of health selection has been addressed in the literature.^11^ Although we controlled for health in childhood, the nature of our independent variable which spans all of adulthood makes developing an appropriate variable to control for adult health complex. More generally, heterogeneity in attitudes, motivations and preferences, like many differences between individuals in social science research, are unobservable.

It is possible that relationships between work-family histories and health may differ according to marital status. If this is the case, then our results may be biased towards the null. Ideally we would have incorporated marital status in the life histories, or included interactions with marital status, because the relationships between work–family histories and health may differ according to marital status. However, this would require a large increase in the number of work–family history categories or generating many interactions, with corresponding reductions in cell sizes which would make null findings more likely.

Despite these limitations, this study has many strengths. Most importantly, we considered patterns of work throughout the lifecourse, from adolescence to after middle age. Sequence analysis allowed us to capture differences in women’s work patterns over many decades. We were able to distinguish between women who had long and short family-related career breaks, and between those who spent the majority of their lives as homemakers/mothers and those who had other reasons for weak attachment to the labour force. Our work–history groupings provide for a parsimonious yet nuanced model that allows the antecedents of later life wellbeing to occur much earlier in life. Due to the richness of the ELSA data we were also able to take account of a number of important covariates.

The associations we report between women’s work and family care histories and their later life health are informative and suggest avenues both for public health intervention and future research. In the short term, work and family histories could be used to identify women at risk of later life disability, allowing targeting of interventions. In the longer term, it is crucial that the mechanisms linking work and family care histories to later life health are better understood. In particular, further research is needed to identify the lifecourse stage at which these group differences emerge. The groups less likely to report disability in later life were also more likely to have good health in childhood, but this selection effect does not fully account for the differences observed. Isolating ‘when’ health trajectories diverge will be an important first step in identifying the underlying causes of these health differences and proposing interventions before retirement age.

## Supplementary data

Supplementary data are available at *EURPUB* online.

## Supplementary Material

Supplementary Figure

Supplementary Data
