# Supplementary figures and images for: Do work and family care histories predict health in older women?
Source: Eur J Public Health. 2017 Sep 23;27(6):1010–5. doi: 10.1093/eurpub/ckx128 (PMC5881779; doi:10.1093/eurpub/ckx128)

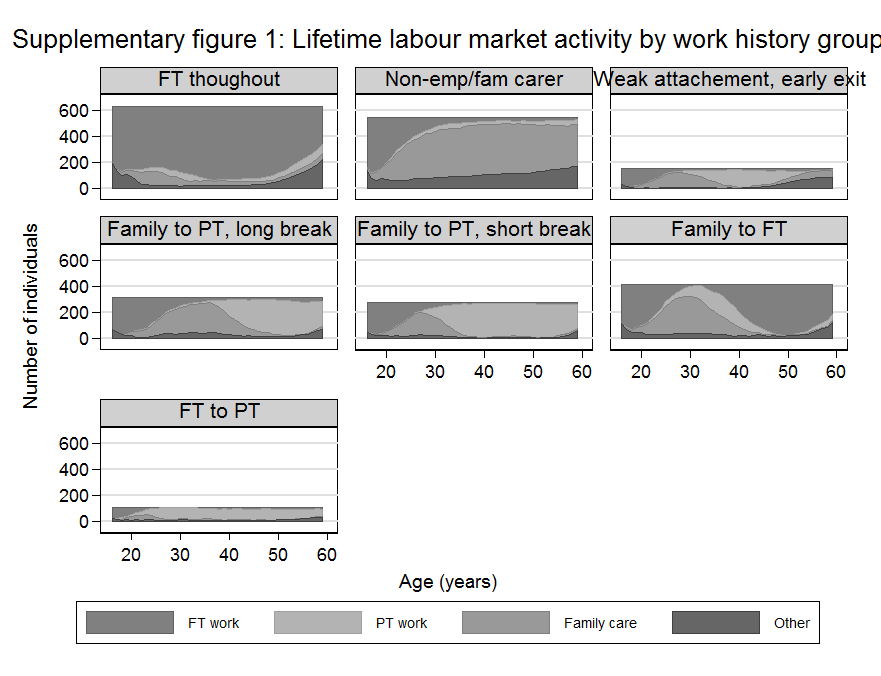

Supplement: Supplementary Figure [file ckx128_ejph-2017-03-om-0185-file003.png]
