# Supplementary material for: Do work and family care histories predict health in older women?
Source: Eur J Public Health. 2017 Sep 23;27(6):1010–5. doi: 10.1093/eurpub/ckx128 (PMC5881779; doi:10.1093/eurpub/ckx128)
Supplement: Supplementary Data [file ckx128_ejph-2017-03-om-0185-file004.docx]

| Supplementary table 1: Characteristics of the sample | | | | | | |  | | | | |  | | | | |  | | | | |  | | | | |  | | | | |  | | | | |  | | | | |  | | | | |  | | | | | | |  | |
| --- | --- | --- | --- | --- | --- | --- | --- | --- | --- | --- | --- | --- | --- | --- | --- | --- | --- | --- | --- | --- | --- | --- | --- | --- | --- | --- | --- | --- | --- | --- | --- | --- | --- | --- | --- | --- | --- | --- | --- | --- | --- | --- | --- | --- | --- | --- | --- | --- | --- | --- | --- | --- | --- | --- | --- |
|  | FTT, children | | | | | FTT, no children | | | | NET, children | | | | | NET, no children | | | | | WA-EE | | | | LB-FT | | | | | SB-FT | | | | | CB-FT | | | | | | PTT | | | | |  | | | Total | | | |  |  |  |  |
|  | % | | | % | | | | | | | % | | | | | % | | | | | % | | | | % | | | | | % | | | | | % | | | | | | % | | | | | p value | | | | | % | | | |  |
| No disability | 71.2 | | | 68.2 | | | | 57.8 | | | | | 64.7 | | | | | 65.0 | | | | | 75.5 | | | | | 83.1 | | | | | 81.7 | | | | | 73.7 | | | | | <0.001 | | | | | | 71.1 | | |  |  |  |  |
| Any disability | 28.8 | | | 31.8 | | | | 42.2 | | | | | 35.3 | | | | | 35.0 | | | | | 24.5 | | | | | 16.9 | | | | | 18.3 | | | | | 26.3 | | | | |  | | | | | | 28.9 | | |  |  |  |  |
|  |  | | |  | | | |  | | | | |  | | | | |  | | | | |  | | | | |  | | | | |  | | | | |  | | | | |  | | | | | |  | | |  |  |  |  |
| Not at risk of depression | 71.1 | | | 69.6 | | | | 61.5 | | | | | 72.5 | | | | | 67.5 | | | | | 69.6 | | | | | 77.3 | | | | | 72.5 | | | | | 64.1 | | | | | 0.001 | | | | | | 69.2 | | |  |  |  |  |
| At risk of depression | 28.9 | | | 30.4 | | | | 38.4 | | | | | 27.5 | | | | | 32.2 | | | | | 30.4 | | | | | 22.7 | | | | | 27.5 | | | | | 35.8 | | | | |  | | | | | | 30.7 | | |  |  |  |  |
|  |  | | |  | | | |  | | | | |  | | | | |  | | | | |  | | | | |  | | | | |  | | | | |  | | | | |  | | | | | |  | | |  |  |  |  |
| Alive February 2012 | 88.7 | | | 84.3 | | | | 84.0 | | | | | 80.4 | | | | | 90.7 | | | | | 94.6 | | | | | 94.5 | | | | | 92.0 | | | | | 88.9 | | | | | <0.001 | | | | | | 89.0 | | |  |  |  |  |
| Dead February 2012 | 11.3 | | | 15.7 | | | | 16.0 | | | | | 19.6 | | | | | 9.3 | | | | | 5.4 | | | | | 5.5 | | | | | 8.0 | | | | | 11.1 | | | | |  | | | | | | 11.0 | | |  |  |  |  |
|  |  | | |  | | | |  | | | | |  | | | | |  | | | | |  | | | | |  | | | | |  | | | | |  | | | | |  | | | | | |  | | |  |  |  |  |
| Father - managerial or professional occupation | 35.1 | | | 35.9 | | | | 34.4 | | | | | 43.1 | | | | | 30.0 | | | | | 31.2 | | | | | 26.3 | | | | | 32.6 | | | | | 21.2 | | | | | 0.047 | | | | | | 32.4 | | |  |  |  |  |
| Father - skilled trade or service occupation | 27.4 | | | 32.7 | | | | 29.0 | | | | | 17.6 | | | | | 35.0 | | | | | 31.9 | | | | | 30.6 | | | | | 30.6 | | | | | 29.3 | | | | |  | | | | | | 30.0 | | |  |  |  |  |
| Father - manual worker | 37.5 | | | 31.3 | | | | 36.6 | | | | | 39.2 | | | | | 35.0 | | | | | 36.9 | | | | | 43.1 | | | | | 36.8 | | | | | 49.5 | | | | |  | | | | | | 37.6 | | |  |  |  |  |
|  |  | | |  | | | |  | | | | |  | | | | |  | | | | |  | | | | |  | | | | |  | | | | |  | | | | |  | | | | | |  | | |  |  |  |  |
| Childhood health - excellent, very good, or good | 86.5 | | | 85.3 | | | | 84.8 | | | | | 74.5 | | | | | 82.1 | | | | | 85.9 | | | | | 88.2 | | | | | 90.2 | | | | | 83.8 | | | | | 0.081 | | | | | | 86.0 | | |  |  |  |  |
| Childhood health - fair or poor | 13.5 | | | 14.7 | | | | 15.2 | | | | | 25.5 | | | | | 17.9 | | | | | 14.1 | | | | | 11.8 | | | | | 9.8 | | | | | 16.2 | | | | |  | | | | | | 14.0 | | |  |  |  |  |
|  |  | | |  | | | |  | | | | |  | | | | |  | | | | |  | | | | |  | | | | |  | | | | |  | | | | |  | | | | | |  | | |  |  |  |  |
| No qualifications | 40.9 | | | 38.7 | | | | 56.8 | | | | | 52.9 | | | | | 45.0 | | | | | 47.3 | | | | | 51.8 | | | | | 36.6 | | | | | 56.6 | | | | | <0.001 | | | | | | 46.9 | | |  |  |  |  |
| Foreign qualifications | 11.5 | | | 12.0 | | | | 12.4 | | | | | 5.9 | | | | | 16.4 | | | | | 11.1 | | | | | 14.1 | | | | | 12.8 | | | | | 15.2 | | | | |  | | | | | | 12.3 | | |  |  |  |  |
| O levels | 14.7 | | | 14.7 | | | | 16.6 | | | | | 19.6 | | | | | 18.6 | | | | | 22.1 | | | | | 17.6 | | | | | 18.0 | | | | | 15.2 | | | | |  | | | | | | 17.1 | | |  |  |  |  |
| A levels | 4.3 | | | 5.5 | | | | 4.4 | | | | | 3.9 | | | | | 2.9 | | | | | 5.0 | | | | | 3.1 | | | | | 6.8 | | | | | 3.0 | | | | |  | | | | | | 4.7 | | |  |  |  |  |
| Any tertiary education | 28.6 | | | 29.0 | | | | 9.8 | | | | | 17.6 | | | | | 17.1 | | | | | 14.4 | | | | | 13.3 | | | | | 25.8 | | | | | 10.1 | | | | |  | | | | | | 18.9 | | |  |  |  |  |
|  |  | | |  | | | |  | | | | |  | | | | |  | | | | |  | | | | |  | | | | |  | | | | |  | | | | |  | | | | | |  | | |  |  |  |  |
| Single, never married | 1.7 | | | 38.2 | | | | 0.4 | | | | | 17.6 | | | | | 0.0 | | | | | 0.0 | | | | | 0.4 | | | | | 0.0 | | | | | 0.0 | | | | | <0.001 | | | | | | 4.2 | | |  |  |  |  |
| Married or cohabiting | 57.2 | | 30.0 | | | | | 58.4 | | | | | 51.0 | | | | | 65.7 | | | | | 68.8 | | | | | 74.1 | | | | | 64.2 | | | | | 64.6 | | | | |  | | | | | | 59.8 | | |  |  |  |  |
| Single, previously married | 41.1 | | 31.8 | | | | | 41.2 | | | | | 31.4 | | | | | 34.3 | | | | | 31.2 | | | | | 25.5 | | | | | 35.8 | | | | | 35.4 | | | | |  | | | | | | 35.9 | | |  |  |  |  |
|  |  | |  | | | | |  | | | | |  | | | | |  | | | | |  | | | | |  | | | | |  | | | | |  | | | | |  | | | | | |  | | |  |  |  |  |
| Never smoker | 37.3 | | 46.1 | | | | | 46.6 | | | | | 49.0 | | | | | 45.0 | | | | | 49.3 | | | | | 52.9 | | | | | 48.4 | | | | | 40.4 | | | | | 0.001 | | | | | | 45.7 | | |  |  |  |  |
| Former smoker | 44.2 | | 40.1 | | | | | 38.6 | | | | | 45.1 | | | | | 40.0 | | | | | 41.9 | | | | | 32.5 | | | | | 39.1 | | | | | 39.4 | | | | |  | | | | | | 39.9 | | |  |  |  |  |
| Current smoker | 18.5 | | 13.8 | | | | | 14.8 | | | | | 5.9 | | | | | 15.0 | | | | | 8.7 | | | | | 14.5 | | | | | 12.5 | | | | | 20.2 | | | | |  | | | | | | 14.5 | | |  |  |  |  |
|  |  | |  | | | | |  | | | | |  | | | | |  | | | | |  | | | | |  | | | | |  | | | | |  | | | | |  | | | | | |  | | |  |  |  |  |
| Wealth quintile 1 (lowest) | 17.3 | | 14.3 | | | | | 22.4 | | | | | 17.6 | | | | | 17.9 | | | | | 12.1 | | | | | 11.8 | | | | | 11.0 | | | | | 20.2 | | | | | 0.053 | | | | | | 16.1 | | |  |  |  |  |
| Wealth quintile 2 | 20.7 | | 16.6 | | | | | 17.4 | | | | | 11.8 | | | | | 20.7 | | | | | 18.1 | | | | | 18.0 | | | | | 20.1 | | | | | 20.2 | | | | |  | | | | | | 18.8 | | |  |  |  |  |
| Wealth quintile 3 | 21.2 | | 20.7 | | | | | 17.0 | | | | | 17.6 | | | | | 21.4 | | | | | 21.8 | | | | | 25.5 | | | | | 20.6 | | | | | 19.2 | | | | |  | | | | | | 20.6 | | |  |  |  |  |
| Wealth quintile 4 | 20.0 | | 24.4 | | | | | 19.8 | | | | | 27.5 | | | | | 20.7 | | | | | 22.8 | | | | | 21.6 | | | | | 23.6 | | | | | 22.2 | | | | |  | | | | | | 21.5 | | |  |  |  |  |
| Wealth quintile 5 (highest) | 20.9 | | 24.0 | | | | | 23.4 | | | | | 25.5 | | | | | 19.3 | | | | | 25.2 | | | | | 23.1 | | | | | 24.8 | | | | | 18.2 | | | | |  | | | | | | 23.1 | | |  |  |  |  |
| Total | 16.9 | | 8.8 | | | | | 20.4 | | | | | 2.1 | | | | | 6.8 | | | | | 14.4 | | | | | 12.3 | | | | | 19.2 | | | | | 4.8 | | | | |  | | | | | |  | | |  |  |  |  |
| N, age 60-75 | | 290 | | | 125 | | | | 271 | | | | | 26 | | | | | 119 | | | | | | | 195 | | | | | 216 | | | | | 295 | | | 92 | | | | |  | | | | | |  | | |  |  |  |
| N, age 76+ | | 126 | | | 92 | | | | 228 | | | | | 25 | | | | | 34 | | | | | | | 120 | | | | | 64 | | | | | 120 | | | 16 | | | | |  | | | | | |  | | |  |  |  |

Note: FTT: mostly full-time throughout; NET: mostly non-employed throughout; WA-EE: weak attachment, early exit; LB-FT: family care to part-time, longer career break; SB-FT: family care to part time, shorter career break; CB-FT: family care to full-time, moderate career break; PTT: mostly part-time throughout.

**
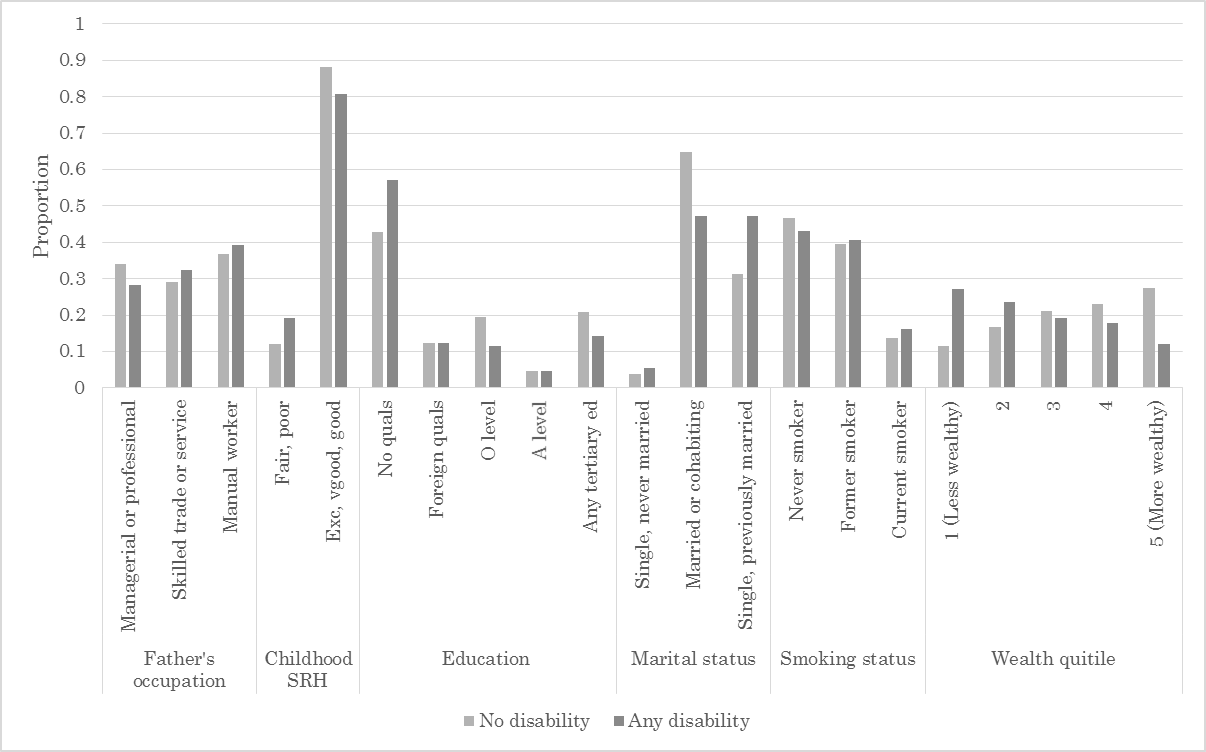
**

**Supplementary figure 2:**  Distribution of covariates by disability status


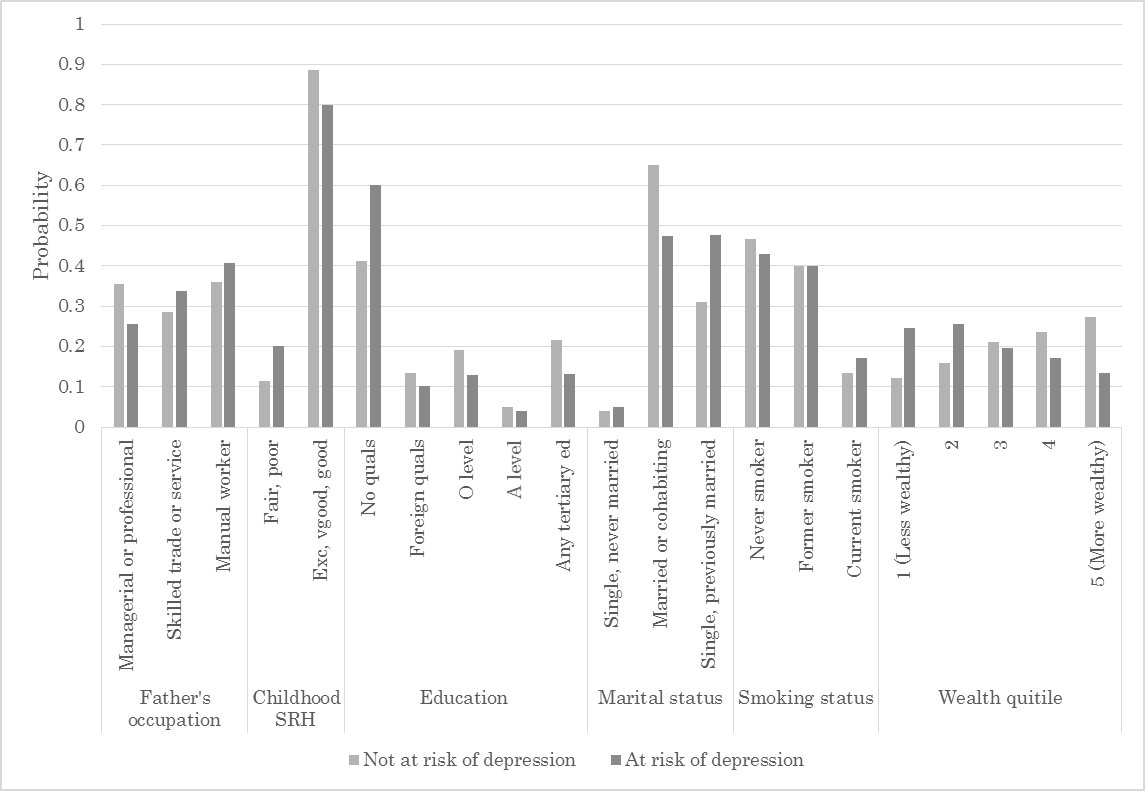


**Supplementary figure 3:** Distribution of covariates by depression status.


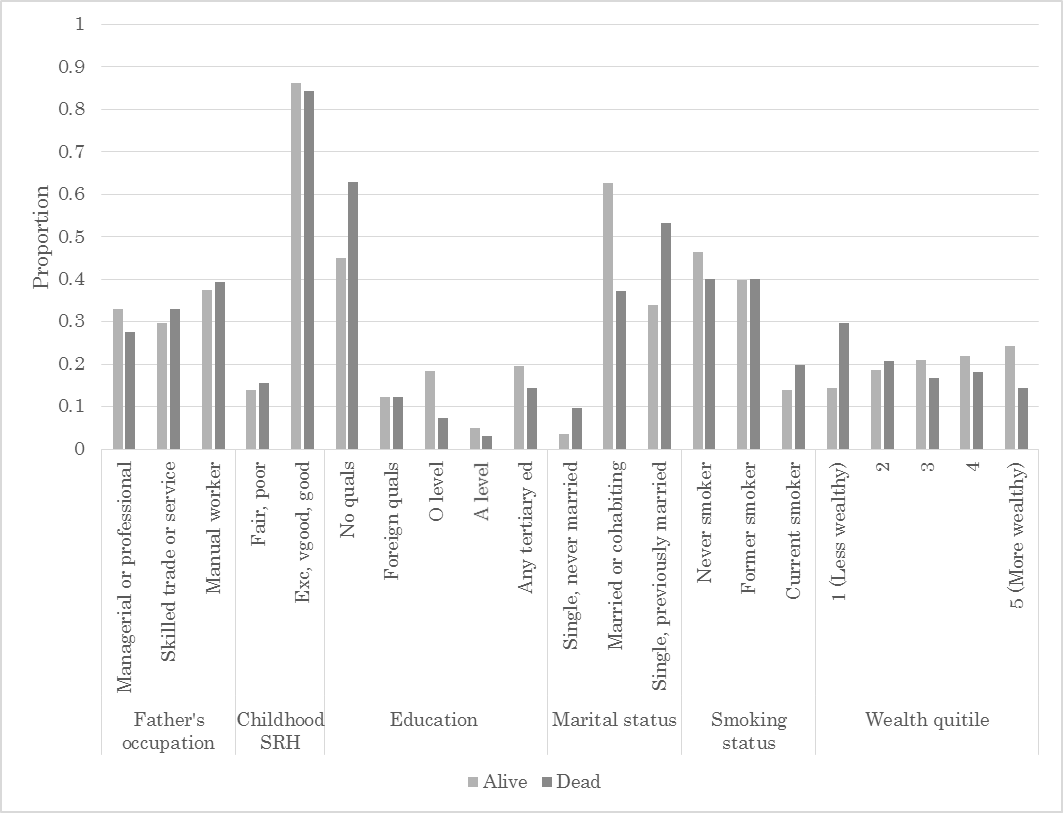


**Supplementary figure 4:** Distribution of covariates by mortality.
